# The palliative role of lasers in the treatment of melanoma

**DOI:** 10.1007/s00403-024-03107-9

**Published:** 2024-05-25

**Authors:** Yanci A. Algarin, Anika Pulumati, Dana Jaalouk, Jiali Tan, Nathalie C. Zeitouni, Keyvan Nouri

**Affiliations:** 1 Eastern Virginia Medical School, Norfolk, VA USA; 2 University of Missouri-Kansas City School of Medicine, Kansas City, MO USA; 3 Florida State University College of Medicine, Tallahassee, FL USA; 4 Albany Medical College, Albany, NY USA; 5 Medical Dermatology Specialists, University of Arizona COM Phoenix, Arizona, USA; 6 Department of Dermatology and Cutaneous Surgery, University of Miami Leonard M. Miller School of Medicine, Miami, FL USA

**Keywords:** Cutaneous melanoma, Laser therapy, Adjunct therapy, Palliative, Metastasis

## Abstract

Melanoma, accounting for a significant proportion of skin cancer-related deaths, has variable survival outcomes based on the stage at diagnosis and treatment efficacy. Traditional treatments, while effective, pose risks of scarring and systemic side effects. Laser therapy offers an emerging non-surgical alternative, with CO2 lasers particularly showing promise in palliative care.

A comprehensive search was conducted using PubMed, focusing on laser therapy for melanoma treatment. The search included studies on both stand-alone and adjunct laser therapies, with inclusion criteria requiring peer-reviewed articles detailing treatment outcomes for primary, recurrent, or metastatic melanoma.

The literature shows that laser therapy for melanoma falls into four major types when categorized by laser medium: solid-state, diode, pulse-dye, and gas (CO2). Data on solid-state lasers for melanoma are limited and their use remains controversial. However, one study with high-energy pulsed neodymium lasers reported a 5-year survival of 82.9% with minimal adverse effects for primary melanoma. CO2 laser therapy has been effective for palliative treatment, with one study showing 54.8% of patients with recurrent melanoma surviving 5.4 years post-ablation. For metastatic melanoma, numerous studies have shown that CO2 laser therapy can provide symptomatic relief and disease control. Combination therapies using lasers and immune-based therapies have demonstrated enhanced outcomes and immune activation, highlighting the potential of laser therapies in melanoma management.

While traditional treatments remain the standard for primary melanoma, laser therapies, particularly CO2 laser ablation, show substantial promise in palliative care for metastatic melanoma. Careful patient selection and assessment are crucial for achieving positive outcomes.

## Introduction

Melanoma is the deadliest form of skin cancer, accounting for 75% of skin cancer deaths despite only accounting for 4% of skin cancer cases [1, 2]. Melanoma accounts for 1.7% of global cancer diagnoses, and is the fifth most prevalent cancer in the United States. Its prognosis is favorable if diagnosed early and surgically excised [3, 4]. Once the melanoma has reached distant metastasis, the survival rate sharply decreases with an estimated 5-year survival rate of 10% [2, 5]. Therefore, early diagnosis and intervention are crucial to patient survival. The treatments for melanoma include surgical excision, radiation therapy, chemotherapy, targeted therapy, and immunotherapy and intratumoral oncolytic viral therapy [2]. While these treatment modalities have shown efficacy, they are accompanied by risks of scarring or systemic adverse effects.

Laser therapy is an emerging non-surgical alternative for the treatment of non-melanoma skin cancers (NMSC). Although some laser therapies have demonstrated efficacy against pre-cancers and various NMSC, its role in melanoma remains controversial and unclear [6]. This review aims to assess the palliative role of lasers in managing primary, recurrent, or metastatic cutaneous melanoma. We will analyze studies that utilize laser therapy, both stand-alone and adjunctive to standard treatments, and evaluate factors such as tumor regression, recurrence rates, overall survival, and adverse effects.

## Methods

### Search strategy

We performed a comprehensive search via PubMed from 1990 to 2023 by utilizing the following search terms and keywords to find relevant articles: “melanoma,” OR “cutaneous melanoma,” OR “melanoma in situ,” AND one of the following search terms: “laser treatment,” OR “laser therapy,” OR “lasers.” The search was restricted to human studies and articles published in English. Additional references were identified from the references of relevant articles.

### Inclusion criteria

The inclusion criteria of our study included articles published in peer-reviewed journals that investigated the treatment of melanoma with laser therapy alone or as an adjunct treatment. The articles needed to contain information regarding treatment outcome, follow-up, recurrence rates, and/or notable adverse events regarding treatment modalities. Articles that did not evaluate lasers for melanoma, lacked sufficient outcome data, and duplicates were excluded.

## Results

### Stand-alone laser therapy

#### Melanoma

##### Solid state lasers

Limited data exists on the use of solid-state lasers for melanoma treatment. One in vitro study assessed the 755 nm alexandrite laser effects on melanoma cell lines, noting increased p16INK4a levels—a protein linked to melanoma progression—in p16INK4a-positive cells post-irradiation [7]. Such laser-induced DNA damage leading to an elevation in p16 expression is undesirable for patients with p16 mutations. Consequently, the researchers cautioned against using lasers for treating melanoma [7].

In an ex vivo study, a 694 nm Q-switched ruby laser (QSRL) was used to treat 42 biopsies from five distinct categories of melanocytic lesions: nevus cell nevi, pigmented dermal nevi, congenital nevi, lentigo maligna lesions, and superficial spreading melanomas [8]. Histology showed selective photothermolysis in pigmented and superficial cells. Lightly pigmented basal layer lesions were ablated with one QSRL treatment, but deeper, heavily pigmented lesions remained. The study advised against QSRL for melanocytic lesions due to potential risks like repigmentation, malignant transformation, or need for repeated treatments, given the limited impact to basal layers and no effect on unpigmented cells [8].

Moskalik et al. retrospectively evaluated the use of two pulsed Neodymium (Nd) lasers (λ = 1060 nm, pulse duration 1 and 4.5 msec, maximum pulse energy 700 and 1000 J, respectively) for the treatment of stage I facial melanoma in 47 patients, delivering 3520–7300 J across 5–12 pulses [9]. After 5 years, the survival rate was 82.9%, with 23.4% developing regional or distant metastases. Lesions were detected in regional lymph nodes in eight (17.0%) and synchronous regional and distant metastases in three (6.4%) patients. No serious adverse effects were reported. It was concluded that high-energy pulsed Nd laser radiation is effective in treating stage I flat and/or raised melanoma lesions of 1–2 mm with Breslow thickness up to 4.0 mm and I-IV Clark levels. However, careful selection of patients for laser treatment is essential to ensure positive results [9]. While these results seem promising, there have been no other studies with the high-energy pulsed Nd laser in treating primary melanoma.

##### Gas - CO2

Gibson et al. evaluated the use of a Sharplan 1030 CO2 laser to treat cutaneous recurrence of melanoma [10]. Forty-two patients with recurrence of lower limb melanoma were identified, with lesions varying from 0.5 to 6 cm in diameter. The median number of lesions was 14 (range: 1–60). A total of 105 treatments were administered, with an average of 2.5 treatments per patient (range 1–9). The median duration between consecutive ablations was 5.2 months (range 1.2–72.0). Among the 42 patients, 19 (45.2%) passed away with a median survival time of 0.8 years from their initial ablation, while the remaining 23 patients (54.8%) had a median survival duration of 5.4 years since their first ablation. Notably, 10 of the 23 (43.5%) of the surviving patients remained free from disease for > 1 year. Complications were minimal with only three wounds taking longer than six weeks to heal. The researchers concluded that while isolated limb perfusion remains their initial treatment of choice for recurrent limb melanoma, CO2 laser ablation is a practical and use approach for palliating local recurrent melanoma [10].

#### Metastatic melanoma

##### Gas - CO2

Kandamany et al. investigated CO2 laser ablation for in-transit cutaneous melanoma in 16 patients with impractical surgical excision due to numerous or recurrent lesions [11]. Using a Coherent UltraPulse 3000 C CO2 laser, 559 lesions were treated. At 12 months post-treatment, 10 (62.5%) patients remained disease-free, while six (37.5%) patients developed extensive disease and succumbed, with a median survival of 1.5 years from initial ablation to death. Long-term remission was observed in six (37.5%) of patients at a median of 7.5 years from the first ablation.

Minimal side effects were noted, with one case of superficial cellulitis. The study supports CO2 laser ablation as a feasible initial palliative approach for melanoma metastases, highlighting its efficiency, cost-effectiveness, and minimal morbidity [11].

Multiple other studies have demonstrated efficacy of CO2 laser therapy as a palliative treatment for cutaneous or regional melanoma metastases [12–17]. A series of investigations utilizing various models of CO2 lasers – including the portable Sharplan 1030, Sharplan 40 C, and Sirius 300 – have treated patients with metastatic melanoma lesions, usually after isolated limb perfusion (IPL) and other treatments have failed. Across these studies, a significant proportion of patients achieved regional disease control, and many survived without recurrence for extended periods (Table 1). Most required fewer than five sessions in the initial year, with median survival ranging from 14 to 45 months, highlighting the potential of CO2 laser therapy in managing melanoma with minimal morbidity. Complications were rare and often related to existing comorbidities. Collectively, these studies suggest that CO2 laser therapy can be a viable primary palliative option for patients with small cutaneous satellite or in-transit lesions where surgery would cause significant morbidity. Palliation with CO2 lasers offers minimal tissue damage, simplified wound management, and favorable cosmetic results [12–16].

**Table 1:** Summary of stand-alone laser and combined laser therapies for melanoma: key findings and outcomes

Source	Study Design	Laser Type	Type of melanoma	Sample size	# of lesions	Follow-up	Treatment regimen (# of sessions, laser settings)	Outcomes	Recurrences	Adverse effects	Quality of Evidence
Solid-State-Laser											
Chan et al.	Experimental, in-vitro	Sub-lethal 755 nm Alexandrite laser	Primary	-	-	-	-	• Increased p16INK4a protein levels following 755 nm Alexandrite laser exposure on melanoma cells • The percentage of HTB 66 cells in the G0/G1 phase of the cell cycle slightly increased with laser fluence, indicating an effect on cell cycle regulation. • Caution against laser use for melanoma.	-	-	5
Kopera et al.	Experimental, ex-vivo	694 nm Q-switched ruby laser (QSRL)	Primary	-	-	-	-	• 694 nm QSRL effectively removed lightly pigmented lesions near the basal cell layer, but heavily pigmented lesions in the dermis persisted. • Caution against QSRL for melanocytic skin lesions.	-	-	5
Moskalik et al.	Retrospective study	1060 nm Nd lasers	Primary (stage I)	47	47 (total)	5–11 years (median 7.5)	Variable, 700 & 1000 J	• 82.9% 5-year survival. • 23.4% developed regional and distant metastases. • 17.0% developed lesions in regional lymph nodes • 6.4% developed synchronous regional and distant metastases.	0	-	3
Gas (CO2)											
Gibson et al.	Retrospective study	Sharplan 1030 CO2 laser	Recurrent (stage III and stage IV)	42	14 per patient (median) (range 1–60)	1–10 years	1–9 sessions, 7–10 W	• 54.8% (*n* = 23) had a median survival duration of 5.4 years since their first ablation. • 10 of the 23 (43.5%) surviving patients remained free from disease for > 1 year.	0	Delayed wound healing (*n* = 3)	3
Kandamany et al.	Retrospective study	Coherent UltraPulse 3000 C CO2 laser	Metastatic (Stage III)	16	559 total	1–10 years	1–14 sessions, 5–30 W	• 62.5%( were disease-free at the 12-month follow-up • A median time of 1.5 years from the first ablation session to death.	0	Superficial cellulitis (*n* = 1)	3
Lingam et al.	Case series	Sharplan 1030 portable CO2 laser	Metastatic (stage III and IV)	19	-	15 months	1–3 sessions, 10–20 W	• 73.7% survived at a mean follow-up period of 15 months. • 42.1% remained free from any recurrence in their limbs.	1	-	4
Van Jarwaarde et al.	Retrospective study	Sharplan 1030 CO2 laser	Metastatic (stage III and IV)	22	643	14 months	1–17 sessions, 7–10 W	• 86.4% of patients achieved regional control lasting a median of 14 weeks (range, 3-117 weeks) • 40.9% achieved control with only a single CO2 laser treatment. • 45.5% required an average of four laser treatments (range, 1–17) to attain regional control. • Median survival of 14 months after the initial laser treatment.	0	Wound infection (*n* = 4)	3
Vrielink et al.	Retrospective Study	Sharplan 40 C CO2 laser	Metastatic (stage III and IV)	26	3 (1–16) per session	5.5 months	1–19) sessions, 5–10 W	• The median survival time after initial treatment was 45 months • 81% of patients received additional treatments, including radiotherapy (33%), systemic treatment (19%), and additional surgical interventions (24%).	-	Delayed wound healing (*n* = 1), ulcer development (*n* = 1)	3
Strobbe et al.	Case series	Sharplan 1030 CO2 laser	Metastatic (stage III)	15	469 (total)	1–9 months	1–5 sessions, 7–9 W	• 46.7% of patients had recurrences at the lasered sites one month post-treatment • 33.3% patients had recurrent in-transit metastases in untreated areas and received additional therapies. • Cautioned against CO2 laser treatment as a first-line option.	7	Delayed wound healing (*n* = 1)	4
Hill et al.	Case series	CO2 laser	Metastatic (stage III, and IV)	60	3–450 (per patient)	30 months	1–18 sessions, 10–20 W	• 56.3% patients with stage IIIa disease had their condition controlled with 3 or less laser treatments within one year	-	Delayed wound healing (*n* = 1)	4
Hill et al.	Case series	Sirius 300 portable CO2 laser	Metastatic (stage II, III, and IV)	100	3–450 (per patient)	1 year	1–10 + sessions, 1–30 W	• 50% of patients with stage IIIa disease were controlled with 3 or less treatments within the first year of follow-up and 64.1% patients by 4 or fewer.	-	Delayed wound healing (*n* = 1)	4
Chan et al.	Case series	UltrapulseCO2 laser (10,600 nm)	Metastatic (III and IV)	3	Variable	2–9 years	1–4 sessions, 60 W	• 66.7% of patients had recurrences. • One patient had liver metastasis before treatment but achieved complete response of cutaneous lesions.	2	Delayed wound healing (*n* = 3)	4
Adjunct Laser Therapy											
Kottschade et al.	Retrospective Study	Pulsed-Dye Laser +/- systemic and/or topical therapy	Metastatic (stage IV)	10	10 (total)	5 months (median)	2–58 sessions, 15 J/cm^2^	• 60% experienced partial local regression • 30% had complete local regression • 10% had no change	1	-	3
Chen et al.	Non-random comparative, controlled clinical trial	Dacarbazine chemo + DNP +/- Laser	Metastatic (stage III and IV)	72	72 (total)	3 years	-	• DNP alone had a 3-year survival rate of 12.2% with a median survival of 19 months. • Combination therapy achieved a 3-year survival rate of 25.9% with a median survival of 28.0 months • 1-year DFS in the monotherapy group was 44.0% • 1-year DFS in the combination therapy group was 69.1%.	0	Fever (*n* = 16), Fatigue (*n* = 27), Neutropenia (*n* = 7), Nausea/Vomiting (*n* = 16), Diarrhea (*n* = 5), Epistaxis (*n* = 2), Increase in BP (*n* = 13), Anemia (*n* = 6)	2
Zeitouni et al.	Case report	Imiquimod + Pulse-Dye Laser	Metastatic (stage IV)	1	50 (total)	6 months	5 sessions, 9 J/cm^2^	• Complete resolution of tumor after treatment for 28 weeks • No recurrence was noted during follow-up	0	-	5
Naylor et al.	Case report	Ipilimumab + 805-nm diode laser	Metastatic (stage IV)	1	4 (total)	12 months	3 sessions, 1 W/cm^2^	• Two small nodules in the right lung disappeared • A larger metastasis in the left lobe persisted but resolved after 9 months of ipilimumab therapy. • PET scan showed complete clearance of all lung metastases one year later	0	-	5
Li et al.	Single-arm phase I/II clinical trial.	ISPI + Diode Laser	Metastatic (Stage III and IV)	11	Variable	72 months	1–6 sessions, 1.0 W/ cm^2^	• Estimated 12-month survival rate was 70% • 72.7% experienced a complete elimination of the tumor from the regional lymphatic drainage area (CLR) • 54.5% had a complete response	0	Fever (*n* = 2), Chills/rigors (*n* = 1), Fatigue/lethargy (*n* = 7), Weight loss (*n* = 4), Dyspnea (*n* = 1), Nausea (*n* = 6), Vomiting (*n* = 1), Anorexia (*n* = 8), Rash (*n* = 10), Pruritus (*n* = 9), Pain (*n* = 7), Cellulitis (*n* = 2)	2

CO2, carbon dioxide; DNP, dinitrophenyl; ISPI, in-situ photoimmunotherapy; PET, positron emission tomography; DSF, disease-free survival; CLR, complete local response

* Study quality assessed using the modified Oxford Centre for Evidence-based Medicine Rating Scale (1 = properly powered and conducted randomized clinical trials or systematic review with meta-analysis; 2 = well-designed controlled trial without randomization or prospective comparative cohort trial; 3 = case-control study or retrospective cohort study; 4 = case series; 5 = opinion of respected authority, case report, or bench research

In contrast to this perspective, Strobbe et al. raised concerns about the suitability of CO2 laser as a first-line treatment for melanoma [18]. They used the Sharplan 1030 CO2 laser at a power of 7–9 W on 15 patients with a total of 469 in-transit and satellite lesions of cutaneous melanoma. There proved to be a high incidence of recurrence in 46.7% of patients approximately one-month post-treatment. They concluded that CO2 laser treatment cannot be considered as a first-line option unless local recurrences are solved. They recommend CO2 laser ablation primarily as a palliative measure after other treatments, emphasizing surgical excision and isolated limb perfusion as initial treatments for limited and extensive limb disease, respectively [18].

The evidence suggests CO2 laser ablation is a viable option for regional disease control in patients with non-resectable satellite or in-transit metastases when surgical resection is not amenable, particularly in the palliative setting. While some argue it should be attempted before IPL [15, 16], most suggest to consider it after other treatment options such as IPL or systemic therapy [12–14].

### Adjunct laser therapy

#### Melanoma

##### Dacarbazine chemo + dinitrophenyl hapten +/- laser

Chen et al. assessed the effectiveness of in situ immunotherapy using dinitrophenyl (DNP) hapten combined with laser therapy in advanced melanoma patients [19]. In the study involving 72 patients with stage lII (in-transit or satellite metastasis) or IV melanoma (distant metastasis), all receiving dacarbazine chemotherapy, the addition of laser therapy to DNP treatment resulted in improved immune response markers and survival outcomes compared to DNP alone. The combination treatment group demonstrated significant increases in interferon-γ levels and reductions in levels of IL-10, TGF-β1, and TGF-β2, correlating with extended overall and disease-free survival. The combination therapy group showed a higher 3-year overall survival rate of 25.9% with a median survival of 28 months, versus 12.2% and 19 months for DNP monotherapy (*P* = 0.024). The combination also led to a 69.1% 1-year disease-free survival rate, exceeding the 44.0% rate of the monotherapy group. No severe adverse side effects were reported, but low-grade fever and fatigue were frequent complaints.

The authors suggest that combining DNP with laser therapy could be a strategic approach to enhance systemic immunity and reduce metastasis in melanoma patients, thereby enhancing the survival of these patients [19]. However, the study is limited by its relatively small sample size, necessitating further validation through multicenter trials with larger populations.

#### Metastatic melanoma

##### Concurrent therapies + pulsed-dye laser

The data available for using pulsed-dye laser (PDL) in treating metastatic melanoma are limited to case reports and series. The largest retrospective case series was published by Kottschade et al., and involved 10 patients with distant metastasis (stage IV disease) [20]. These patients received PDL therapy, with or without imiquimod and/or systemic treatment. The median duration of PDL therapy was 5 months (range 2–29). Six patients (60%) experienced partial local regression, three patients (30%) had complete local regression, and one patient (10%) showed no change. Notably, five out of six patients who experienced partial local regression developed systemic disease progression. Despite varied outcomes, the study found a significant improvement in morbidity and life quality in patients treated with PDL, and recommend it as a viable and safe palliative treatment for those ineligible for additional surgical removal of skin metastases [20].

##### Imiquimod + pulse-dye laser

Zeitouni et al. reported on an 82-year-old male patient with 50 + melanoma metastases on the right thigh and lower leg treated with combination topical imiquimod 5% and PDL [21]. Initially, he began the treatment with imiquimod 5% three times weekly. At four weeks, the frequency was increased to daily application in conjunction with one session of 595-nm wavelength PDL (7 mm spot size, 9 J cm^− 2^). At week six, the lesions were visibly reduced and at eleven weeks, more than half of the lesions were resolved. The patient continued imiquimod treatment for 28 weeks and received a total of 5 PDL sessions. Biopsies post-treatment confirmed no melanoma presence, with a clear 6-month follow-up [21]. These results indicate the potential of PDL to enhance the anti-tumor effects of imiquimod therapy. Nevertheless, additional studies involving larger sample sizes are needed to determine the true efficacy of combination therapy with imiquimod and PDL.

##### Ipilimumab + 805-nm diode laser

Currently, there are no reports on the use of stand-alone diode lasers for the treatment of metastatic melanoma. One case report involving a 65-year-old male with stage IV melanoma metastasis to his head, neck, and lungs explored the use of laser immunotherapy (LIT) following unresponsiveness to surgical and radiation treatments [22]. The treatment regimen included an 805-nm diode laser applied at 1 W/cm^2^ for 10 min across three sessions bi-weekly, complemented by twice-daily topical imiquimod 5%. Post three months of LIT, the patient was administered a four-dose course of ipilimumab (3 mg/kg) at three-week intervals, which was well-tolerated. Subsequent imaging revealed resolution of two small right lung nodules and, after 9 months, resolution of a larger left lung metastasis. A year after starting ipilimumab, a PET scan confirmed the absence of lung metastases, highlighting the potential synergistic effect of LIT combined with systemic immunotherapy in treating melanoma; however, more studies with larger sample sizes are needed to confirm [22].

##### In-situ photoimmunotherapy + diode laser

Li et al. conducted a clinical trial on 11 patients with stage III and IV melanoma, administering In-Situ Photoimmunotherapy (ISPI) that combined an 805 nm diode laser, imiquimod, and indocyanine green (ICG) injections. Patients underwent one or more 6-week cycles of treatment on a 200-cm2 area. All the subjects completed at least one treatment cycle.

Significant symptomatic relief in the treated regions was reported by all patients. Moreover, all treated melanoma lesions responded to ISPI, with 72.7% of patients achieving complete local response (CLR) and 54.5% showing a complete response (CR). CLR was also noted in untreated regions in some patients, suggesting systemic immune activation. The 12-month survival rate post-ISPI was 70%, with manageable side effects such as rash and pruritus being the most common. No severe toxicity was reported. ISPI, which is administered on an outpatient basis and compatibility with other treatments, was favored by patients over chemotherapy for its tolerability and less detriment to their quality of life. The study concluded that ISPI using a diode laser and imiquimod is a safe and useful palliative modality for advanced melanoma [23].

## Discussion

Laser therapy for melanoma falls into four major types when categorized by laser medium: solid-state, diode, pulse-dye, and gas. Although, several interventions have shown promise in treating primary, recurrent, or metastatic cutaneous melanoma, CO2 laser stands as the most utilized and effective palliative treatment for melanoma. Still, methodological variations within laser therapies often determine their success or lack thereof.

In the context of stand-alone laser therapy, limited data are available on the efficacy of solid-state lasers and PDL for melanoma treatment. The Nd laser has shown promising results for stage I cutaneous melanoma, reporting a 5-year survival of 82.9% with minimal adverse effects [9]. However, other studies raised concerns about the potential impact of solid-state laser exposure on melanoma cells, particularly the sub-lethal 755 nm alexandrite laser and QSRL, and recommended against their use for melanoma treatment [7, 8]. This uncertainty underscores the need for caution when selecting solid-state lasers as a primary treatment modality for melanoma. Currently, the Nd laser should not be recommended for primary melanoma, as more studies with larger patient populations are needed to establish its efficacy and establish treatment protocols. No study has evaluated solid-state lasers for the treatment of metastatic melanoma. In the case of PDL, the limited data are largely based on case series for treating metastatic melanoma [20]. The results were variable, with some patients experiencing partial or complete regression of the metastatic lesions, and some no response. Most notable, however, was that majority of patients treated experienced enhanced quality of life and reduced morbidity. While randomzed controlled trials (RCT) are needed to determine the true efficacy of PDL lasers for melanoma, the results show that PDL lasers with adjunct topical or systematic therapy can be a valuable palliative option for patients with metastatic melanoma who cannot undergo surgical resection.

However, the use of CO2 laser ablation for melanoma has shown promising results across various studies [10–17]. With regards to stand-alone treatment for recurrent melanoma (*n* = 42), 54.8% of patients had a median survival duration of 5.4 years since their first ablation. In several patients, complete remission of the disease was achieved, and ten patients were disease-free for more than 1 year [10]. Additionally, the studies on CO2 laser ablation for cutaneous metastases in melanoma patients consistently demonstrate its effectiveness as a therapeutic and palliative intervention [11–16]. Surgical resection remains the standard for managing limited satellite or in-transit melanoma metastases, but may be impractical for head and neck locations or widespread disease due to the risk of disfigurement [24–26]. In cases of advanced melanoma where surgery is not feasible, has previously failed, or is declined by the patient, CO2 laser ablation can be used for palliation or to establish regional control in select patients with unresectable in-transit disease [11–16].

Combining laser therapy with other treatment modalities, such as immunotherapy and topicals, provides a more nuanced approach for managing melanoma. Studies have shown that several different laser and topical combinations can be effective in managing primary [19] and metastatic melanoma [20, 22, 23]. For primary melanoma, DNP treatment combined with laser immunotherapy was the only combined treatment modality found in the literature. The combination therapy group had a significantly higher 3-year OS rate of 25.9% and a median survival of 28.0 months(*P* = 0.024). Additionally, the 1-year DFS in the combination therapy group was 69.1% in contrast to that of the monotherapy group at 44.0%. For metastatic melanoma, two case reports have explored the combination of laser therapy with immunomodulators, such as imiquimod [21], and immunotherapies such as ipilimumab [22]. These combinations showed encouraging results, with both patients experiencing complete tumor regression, symptom relief, and enhanced immune responses [21, 22]. Notably, combining ISPI with an 805 nm diode laser and imiquimod demonstrated a high rate of complete local responses (72.7%) and complete responses (54.5%) in advanced melanoma cases. The reported adverse events were generally manageable, making ISPI a relatively well-tolerated outpatient procedure. These findings highlight the potential of laser therapies to complement the existing approaches for managing melanoma. However, consistent with Karrera et al., we do not endorse laser therapy for primary malignant melanoma at any stage due to the insufficient evidence base. RCT with larger sample sizes are needed to further establish the role of laser therapy in managing primary melanoma.

The use of lasers in melanoma treatment is not standard and is often tailored to the individual patient’s situation. The literature suggests that offering laser treatment can be a valuable option for patients with metastatic melanoma who are not viable surgical candidates and/or prefer a palliative approach [11, 13]. Importantly, standard treatment approaches for patients with metastatic melanoma should first be considered such as intralesional injections, adjunct radiation, or immunotherapy [27]. Currently, there are no protocols and referral criteria for laser treatment in melanoma. While the National Comprehensive Cancer Network’s cutaneous melanoma guidelines categorize CO2 laser ablation as a Category 2B palliative treatment, they do not provide detailed recommendations regarding the selection of CO2 laser types, nor the optimal settings and treatment durations [28]. Therefore, individualized treatment planning, guided by clinical judgment and patient-specific factors, is essential for achieving positive outcomes with CO2 laser ablation in this context. Because the realistic aim of treatment is not to cure, a major benefit of laser ablation is that it can be performed with little disruption to the patient’s life. In addition, the low rate of complications associated with laser ablation makes it a viable alternative for controlling recurrent melanoma, offering a balance between efficacy and safety. More importantly, as a palliative treatment this approach may provide patients with a higher level of comfort and extend the duration of relief from their symptoms. However, it is important to appreciate that laser treatment is not suitable for all patients, particularly those with deep subcutaneous lesions, ulcerated lesions, and lesions larger than 2 cm [16].

The studies reviewed varied in sample size, methodology, and patient populations, which can influence the generalizability of the results. RCT and larger-scale studies are warranted to substantiate the findings. Specifically, there is a need for more systematic investigations of adjunctive laser therapies with well-defined controls for confounding factors such as medication concentrations, photosensitizers, treatment frequency, and intervals between follow-up and test-of-cure evaluations. Additionally, the long-term safety and efficacy of laser therapy in melanoma management remains uncertain, emphasizing the need for larger patient cohorts and RCT. Finally, several reports have identified melanomas being identified within lesions that had previously received laser therapy [29–34]. Thus, it is crucial to ensure that the diagnosis is biopsy-proven before proceeding with laser therapy. In addition, patients need to be informed that lasers can miss deeper-lying melanocytes and subsequent recurrences [29].

## Conclusion

The role of laser therapy in the management of melanoma represents a nuanced and evolving area of dermatologic oncology. The goal of laser treatment in this context is not curative but rather palliative, aiming to minimize symptoms and improve the patient’s daily experience while maintaining a low complication profile. The cautious optimism surrounding laser therapy is warranted; however, it is accompanied by a responsibility to ensure that patients are thoroughly educated about their options. Surgical intervention remains the standard of care primary melanoma, however laser therapy can be considered for metastatic melanoma for patients who are poor surgical candidates and/or prefer a palliative approach. Adjunct radiation, intralesional injections, or immunotherapy should still be considered first. Current literature reinforces the need for larger, well-designed studies to establish standardized protocols and to further evaluate the long-term safety and efficacy of laser therapies in melanoma.

## Data Availability

Not applicable.
